# Factors involved in initiation and regulation of complement lectin pathway influence postoperative outcome after pediatric cardiac surgery involving cardiopulmonary bypass

**DOI:** 10.1038/s41598-019-39742-w

**Published:** 2019-02-27

**Authors:** Mateusz Michalski, Izabela Pągowska-Klimek, Steffen Thiel, Anna S. Świerzko, Annette G. Hansen, Jens C. Jensenius, Maciej Cedzyński

**Affiliations:** 1 Laboratory of Immunobiology of Infections, Institute of Medical Biology, Polish Academy of Sciences, Lodz, Poland; 2 Department of Pediatric Anesthesiology and Intensive Care, Medical University of Warsaw, Warsaw, Poland; 3 Department of Biomedicine, Aarhus University, Aarhus, Denmark

## Abstract

Congenital heart disease (CHD) often requires surgical intervention, and is sometimes associated with life-threatening post-operative complications. We have investigated some factors of the innate immune system involved in the initiation or regulation of complement lectin pathway activation (MASP-1, MASP-2 MASP-3, MAp19, MAp44, ficolin-3) and related them to complications and prognosis in 190 pediatric patients undergoing CHD repair with the use of cardiopulmonary bypass (CPB). Patients with MAp44 levels ≤1.81 µg/ml more frequently experienced low cardiac output syndrome (LCOS), renal insufficiency, systemic inflammatory response syndrome (SIRS) and multiorgan dysfunction (MODS). Low MASP-3 (≤5.18 µg/ml) and high MASP-1 (≥11.7 µg/ml) levels were often associated with fatal outcome. Low ficolin-3 concentrations (≤10.1 µg/ml) were more common among patients experiencing SIRS and MODS than in those without complications. However, patients suffering from SIRS and MODS with low ficolin-3 had a much better prognosis (91% survival *vs*. 37% among other patients; p = 0.007). A discriminating value of 12.7 µg/ml ficolin-3 yielded 8% *vs*. 60% mortality (p = 0.001). Our data extend the knowledge concerning involvement of proteins of the lectin pathway in development of post-CPB complications. The potential prognostic value of low preoperative MAp44 and high preoperative ficolin-3 seems promising and warrants independent confirmation.

## Introduction

Congenital heart disease (CHD) is a leading cause of infant mortality^1^. Its incidence differs among various populations, but generally, it is estimated as 10–12/1000 live births, corresponding to approx. 1.3 million cases worldwide yearly^2^. Some less severe defects may be consistent with relatively good health and a normal life, but others require surgery (approx. 25%) or other interventions during the first year of life. Some patients suffer from life-threatening post-operative complications, prolonging intensive care unit stay and increasing costs of the therapy^3^. Patients may be affected by hospital-acquired infections, low cardiac output syndrome (LCOS) and systemic inflammatory response syndrome (SIRS) which may further lead to multiple organ dysfunction syndrome (MODS). The inflammation occurring after cardiac surgery and cardio-pulmonary bypass (CPB) involves multiple cellular and humoral pathways, among which the complement system plays a central role^4^. Three major complement activation pathways have been described: the classical (CP), the alternative (AP) and the lectin pathway (LP). The last is activated by specific pattern-recognition molecules (PRMs), *i*.*e*., ficolins and some collectins, complexed with enzymes belonging to the MASP (mannose-binding lectin serine protease) family. The involvement of the mannose-binding lectin (MBL)-dependent lectin pathway activation in the inflammatory response following CPB has been documented by us previously^5,6^. Therefore we decided to investigate the significance of particular MASPs and related non-enzymatic (regulatory) proteins. Furthermore, we investigated ficolin-3 (H-ficolin) as the most abundant lectin pathway-associated PRM.

Serine proteases (MASP-1, MASP-2, MASP-3) and non-enzymatic MAp44 (also termed MAP-1) and MAp19 (or sMAP) are in turn complexed with ficolins/collectins, enabling an initiation of complement activation, its regulation and/or cross-talk with other cascades like coagulation system. MASP-1, MASP-3 and MAp44 are products of alternative splicing of RNA of the *MASP1/3* gene whereas MASP-2 and MAp19 are splice variants of RNA of the *MASP2* gene^7,8^. PRM-MASP complexes are considered important players of the first-line antimicrobial innate immune defense. Beside microbial structures, their activation can be triggered by apoptotic cells, necrotic debris or other aberrant self-structures^9^. Auto-activated MASP-1 is responsible for MASP-2 activation as well as for cleaving of C4-bound complement component C2^10^. MASP-2 activates C4 and C4-bound C2^11^. A substrate for the MASP-3 protease is profactor D, which leads to generation of factor D, an enzyme involved in the initiation of the alternative pathway of complement^12,13^. On the other hand, MASP-3 has also been suggested to hinder the LP cascade^7,14^. Similar inhibitory activity was proposed also for MAp44^15^. Although MAp19 was thought to be a LP regulatory factor as well, its biological significance remains to be clarified, since in physiological conditions it did not influence C4 cleavage product deposition^16^. MASP-1 and MASP-2 may contribute to thrombogenesis from their ability to cleave fibrinogen, factor XIII, prothrombin and thrombin activatable fibrinolysis inhibitor (TAFI)^17–19^.

Factors influencing MASP/MAp and ficolin-3 synthesis are still unknown, however major surgery was associated with a drop in ficolin-3, MASP-1, -2, -3 and MAp44 concentrations within 12–48 h^20–23^. However, in contrast to MASP-3, a marked increase of MASP-1 and MAp44 within 4–20 post-operative days was observed, suggesting they could be acute phase proteins^20,21^. Furthermore, MASP-2 was suggested to be an acute-phase reactant based on data from patients with acute pancreatitis^24^.

Here we report an investigation of pre-operative serum MASP-1, MASP-2, MASP-3, MAp44, MAp19 and ficolin-3, concentrations and their possible influence on the incidence of post-operative complications in infants and children operated on because of congenital heart disease. As our study has strictly been focused on the understanding of involvement of complement lectin pathway in complications after CHD repair, we have not recruited healthy controls.

## Material and Methods

### Patients

One hundred and ninety patients (77 girls and 113 boys), aged from 3 months to 17 years (mean: 3 years and 4 months), undergoing primary cardiac surgery for the reason of CHD, with the use of CPB were recruited. Seventy-nine patients (aged from 3 to 12 months) were defined as infants while 111 were defined as older children. There were no differences in male/female ratio between age groups.

The types of CHDs diagnosed as well as other clinical and demographic data are listed in Table 1. Basic Aristotle Score (BAS) was used for evaluation of surgical procedure complexity, potential for mortality and morbidity as well as technical difficulty^25^. Exclusion criteria were: need for pre-operative mechanical ventilation, pre-operative infection or organ dysfunction, and death during surgery. The post-operative course was observed and documented until hospital discharge. Patients were screened for symptoms of post-operative complications (infection, SIRS, LCOS, organ dysfunctions), essentially as described previously^5,6^.

**Table 1:** Characteristics of patients

	Infants	Children	p
	79	111	
	33/46	44/67	0.93
**Age (years)**			
mean	0.62	5.29	<0.0001
median	0.5	5	
range	0.1–1	1.5–17	
**Basic Aristotle Score (BAS)**			
mean	7.02	8.00	<0.0001
median	7	9	
range	3–11	3–13.8	
**Single ventricle anatomy**			
n (%)	27 (34.2)	50 (43.5)	0.19
**Congenital heart defect (CHD) n (%)**			
atrial septal defect	7 (8.9)	14 (12.2)	0.47
ventricular septal defect	19 (24.1)	8 (7.0)	0.001^a^
tetralogy of Fallot	12 (15.2)	13 (11.3)	0.43
hypoplastic left heart syndrome	14 (17.7)	32 (27.8)	0.1
hypoplastic right heart syndrome	2 (2.5)	2 (1.7)	0.7
tricuspid atresia	3 (3.8)	8 (7.0)	0.35
pulmonary valve atresia	0 (0.0)	7 (6.1)	0.026^b^
mitral valve atresia	4 (5.1)	1 (0.9)	0.07
atrioventricular septal defect	8 (10.1)	4 (3.5)	0.059
double inlet left ventricle	1 (1.3)	2 (1.7)	0.79
double outlet right ventricle	2 (2.5)	4 (3.5)	0.71
transposition of the great arteries	2 (2.5)	3 (2.6)	0.97
aortic stenosis	1 (1.3)	8 (7.0)	0.064
others	4 (5.1)	13 (11.3)	0.13
**Vasoactive Inotropic Score (VIS)**			
mean	12	25	0.0002
median	5	12	
range	0–100	0–100	
**Length of the stay at ICU (days)**			
mean	5.8	5	0.35
median	2	2	
range	1–84	0–75	
**Length of the stay at hospital (days)**			
mean	13	15	0.009
median	9	13	
range	0–95	0–89	
**Post-operative complications n (%):**			
Fever	15 (19.0)	26 (22.6)	0.54
Infection	18 (22.8)	21 (18.3)	0.44
SIRS	14 (17.7)	28 (24.3)	0.27
SIRS + MODS	8 (10.1)	22 (19.1)	0.088
Low Cardiac Output Syndrome (LCOS)	9 (11.4)	25 (21.7)	0.063
Renal insufficiency	3 (3.8)	15 (13.0)	0.029^c^
Liver failure	6 (7.6)	22 (19.1)	0.025^d^
Death	6 (7.6)	11 (9.6)	0.63

^a^OR = 0.24, 95% CI (0.1–0.57); ^b^OR = 10.99, 95% CI (0.62–195.4); ^c^OR = 3.81, 95% CI (1.06–13.6); ^d^OR = 2.88 95% CI (1.11–7.47).

Cardiovascular status was assessed using the vasoactive inotropic score (VIS)^26^. SIRS was diagnosed according to Goldstein *et al*.^27^, and multiorgan dysfunction (MODS) when dysfunction of at least two organs was observed. Low cardiac output syndrome (LCOS) was defined according to clinical criteria including: tachycardia, poor peripheral perfusion, hypotension, need for introducing new inotropic agent or increase doses twice, oliguria, cardiac arrest, elevated lactates. Hepatic dysfunction was defined as prothrombin time at least 2x normal and ALT > 100 IU/l; renal insufficiency was defined as the need for renal replacement therapy. The approval of the Bioethical Committee of the Polish Mother’s Memorial Hospital Research Institute and written informed parental consent were obtained. This work conforms to the provisions of the Declaration of Helsinki.

### Clinical material

Serum samples were obtained from blood taken in the operating room just before cardiac surgery into tubes without anticoagulant^5,6^ and then kept at 4 °C for 30 min for clotting, centrifugated, distributed to Eppendorf tubes and stored at −80 °C.

### Determination of ficolin-3 and MASP family proteins serum concentrations

Serum levels of the MASP family proteins were determined using assays described elsewhere: for MASP-1^28^, MASP-2^29^, MASP-3^20^, MAp44^20^ and MAp19^16^. Ficolin-3 concentrations were measured by ELISA as described previously^30^ with later modification^29^. For some analyses, cut-off values out of interquartile range (IQR; lower or higher) were chosen arbitrarily. They are presented in Table S1.

### Statistical analysis

The Shapiro-Wilk test was used to determine normality. As the distribution of pre-operative levels of tested proteins was not normal (see below), the medians were compared using the Mann-Whitney *U*-test. The frequencies of low or high protein concentrations, as well as post-operative complications were compared by χ2 test or χ2 test with Yates correction (depending on expected value) and in smaller groups (n < 40) by two-sided Fischer’s exact test. Correlations were determined by Spearman’s test. P values < 0.05 were considered statistically significant.

## Results

### Basic clinical data

The types of CHDs diagnosed as well as other clinical and demographic data are listed in Table 1. The most common procedures were: bidirectional Glenn, Fontan operation, Ross operation, correction of tetralogy of Fallot, ventricular septal defect (VSD) repair, atrial septal defect repair, atrioventricular canal (CAV) defect repair. Mean CPB time was 85.9 minutes (±42.4) and mean aorta cross-clamping time was 42.9 min (±27.2).

The Basic Aristotle Score, reflecting surgery technical difficulties and the risk of complications, was significantly higher in the older children (p < 0.0001). They also more often required pharmacological cardiovascular support (VIS) after surgery (p = 0.0002) and were more frequently diagnosed with post-operative renal insufficiency [OR = 3.81, 95% CI (1.06–13.6), p = 0.029] or liver failure [OR = 2.88, 95% CI (1.11–7.47), p = 0.025] in comparison to infants and they needed markedly longer stays in hospital (p = 0.009).

### Pre-operative concentrations of lectin pathway-associated proteins in infants and older children

**Figure 1:**
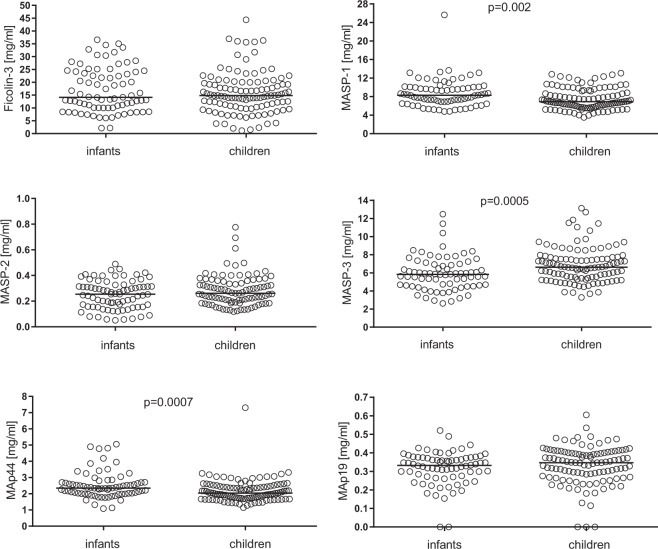
The pre-operative serum concentrations of ficolin-3, MASP-1, MASP-2, MASP-3, MAp44 and MAp 19 in infants and children.

The pre-operative serum concentrations of MASP-1, MASP-2, MASP-3, MAp44, MAp19 and ficolin-3 were estimated in both groups of patients (Fig. 1). MASP-1 and MAp44 were slightly but significantly higher in infants while the opposite was true for MASP-3. Consistently with this, MASP-3 correlated positively while MASP-1 and MAp44 – inversely, with age. Serum MAp44 significantly correlated with the majority of other proteins tested (except for MASP-2), and most strongly with MASP-1 (Table S2).

Serum MAp44 and MASP-3 concentrations correlated inversely with surgical complexity (BAS) in older children, whereas Map44, MASP-3 and MAp19 correlated inversely with the need for pharmacological cardiovascular support (VIS) (Table 2).

**Table 2:** Correlations of lectin pathway-associated proteins with parameters of disease severity, depending on patient’s age.

Protein	Clinical parameter			
	BAS		VIS	
	Infants	Children	Infants	Children
Ficolin-3	r = −0.02; p = 0.9	r = 0.02; p = 0.84	r = −0.02; p = 0.86	r = −0.02; p = 0.85
MASP-1	r = −0.05; p = 0.68	r = −0.13; p = 0.19	r = −0.05; p = 0.69	r = 0.03; p = 0.78
MASP-2	r = 0.06; p = 0.61	r = 0.03; p = 0.78	r = 0.14; p = 0.22	r = 0.02; p = 0.81
MASP-3	r = −0.09; p = 0.47	**r = −0**.**27; p = 0**.**005**	r = −0.05; p = 0.65	**r = −0**.**21; p = 0**.**03**
MAp44	r = −0.22; p = 0.057	**r = −0**.**23; p = 0**.**02**	r = 0.07; p = 0.54	**r = −0**.**21; p = 0**.**03**
MAp19	r = −0.18; p = 0.13	r = −0.14; p = 0.17	r = 0.09; p = 0.47	**r = −0**.**24; p = 0**.**01**

### Pre-operative concentrations of lectin pathway-related proteins and post-operative complications

To analyze the possible associations of pre-operative concentrations of lectin pathway factors with post-operative complications, data from patients with such events as fever, infections, SIRS, SIRS accompanied with MODS, LCOS, liver failure, renal insufficiency, and those without complications were compared. Furthermore, the results from survivors and non-survivors were analyzed. No significant differences in patient’s age or BAS were noted between the subgroups being compared (not shown). On the other hand, median VIS was significantly higher in patients suffering from each of those post-operative complications, in comparison with the reference group (p < 0.0001). Moreover, as expected, the stay at ICU and in the hospital in general, was significantly longer in patients with post-operative complications (not shown).

### MASP-1, MASP-2 and MASP-3

Median MASP-1, MASP-2 and MASP-3 concentrations in patients with no complications were 7.5 µg/ml, 0.25 µg/ml and 6.2 µg/ml, respectively (Table 3). MASP-3 levels were significantly lower in patients who died as a result of post-operative complications in comparison with survivors (5.4 μg/ml *vs*. 6.3 μg/ml, p = 0.027). Moreover, patients with low MASP-3 (<5.18 µg/ml) had generally a worse prognosis than those with higher values [22.2% *vs*. 7.2% deaths (OR = 3.67, 95% CI, 1.04–12.9)].

**Table 3:** Serum pre-operative concentrations (median, interquartile range) and incidence of post-operative complications.

	Ficolin-3	p	MASP-2	p	MAp19	p
	median (IQR)[µg/ml]		median (IQR)[µg/ml]		median (IQR)[µg/ml]	
No complication	14.86 (10.82–21.49)		0.25 (0.18–0.36)		0.36 (0.29–0.4)	
SIRS	13.24 (8.29–19.53)	0.16	0.27 (0.19–0.33)	0.94	0.34 (0.25–0.37)	0.14
SIRS + MODS	12.79 (7.12–17.13)	0.07	0.24 (0.19–0.39)	0.57	0.31 (0.23–0.38)	0.069
infection	16.64 (10.01–22.58)	0.68	0.30 (0.22–0.40)	0.15	0.33 (0.25–0.38)	0.19
fever	13.09 (8.06–20.34)	0.22	0.22 (0.15–0.32)	0.28	**0**.**33** (**0**.**22–0**.**37)**	**0**.**02**
LCOS	13.77 (8.22–22.66)	0.36	0.25 (0.19–0.34)	0.86	0.31 (0.23–0.4)	0.24
liver failure	12.48 (7.95–19.64)	0.11	0.22 (0.18–0.36)	0.69	0.31 (0.24–0.38)	0.057
renal insufficiency	13.89 (8.68–18.16)	0.42	0.22 (0.18–0.36)	0.77	0.33 (0.28–0.39)	0.49
death	14.84 (12.8–22.59)	0.76	0.29 (0.20–0.41)	0.31	0.35 (0.31–0.41)	0.73
	**MASP-1**	**p**	**MASP-3**	**p**	**MAp44**	**p**
	**median** (**IQR)[µg/ml]**		**median** (**IQR)[µg/ml]**		**median** (**IQR)[µg/ml]**	
No complication	7.52 (6.13–9.74)		6.22 (5.21–7.67)		2.33 (1.96–2.66)	
SIRS	7.05 (5.75–8.67)	0.3	5.88 (4.85–6.69)	0.092	**1**.**94** (**1**.**68–2**.**32)**	**0**.**004**
SIRS + MODS	7.05 (5.34–9.41)	0.34	5.92 (5.11–6.62)	0.17	**1**.**85** (**1**.**66–2**.**18)**	**0**.**003**
infection	7.44 (6.01–9.97)	0.77	6.45 (5.17–7.53)	0.88	2.11 (1.74–2.53)	0.28
fever	7.39 (5.61–9.44)	0.69	5.93 (5.06–7.29)	0.32	2.06 (1.79–2.42)	0.14
LCOS	7.05 (5.5–9.68)	0.61	5.94 (5.11–6.81)	0.43	**1**.**92** (**1**.**67–2**.**45)**	**0**.**03**
liver failure	6.99 (5.53–9.41)	0.45	5.94 (5.34–7.05)	0.42	**1**.**92** (**1**.**67–2**.**17)**	**0**.**005**
renal insufficiency	7.25 (5.58–9.84)	0.83	5.95 (5.07–6.57)	0.16	**1**.**74** (**1**.**64–2**.**19)**	**0**.**004**
death	8.48 (6.33–11.72)	0.26	5.4 (4.05–6.51)	0.037	2.19 (1.65–2.75)	0.58

Additionally, children with the highest levels of MASP-1 (≥90^th^ percentile, 11.68 µg/ml), had a much lower rate of survival compared with individuals with lower MASP-1 concentrations. No such relationship was found in the case of MASP-2 (data not shown).

### MAp44 and MAp19

Median pre-operative concentrations of MAp44 and MAp19 among patients who did not suffer from post-operative complications were 2.33 µg/ml and 0.36 µg/ml, respectively (Table 3). In the case of MAp44, the median was significantly lower in patients experiencing post-operative LCOS, SIRS and/or organ dysfunctions whereas lower MAp19 was associated with patients with fever (Table 3). Furthermore, for older children, lower MAp19 levels were associated with SIRS, (accompanied or not with multiorgan dysfunction), single organ dysfunctions and LCOS (not shown).

These relationships were confirmed by analysis of low serum concentrations of MAp44 and MAp19 (Table 4). The patients with low MAp44 more frequently experienced post-operative LCOS, renal insufficiency, SIRS and SIRS + MODS. Low MAp19 concentrations were markedly more common among patients with fever.

**Table 4:** Frequencies of low (≤interquartile range) pre-operative concentrations of investigated proteins, depending on incidence of post-operative complications.

complications	Ficolin-3 ≤ 10.1 µg/ml		MASP-2 ≤ 0.18 µg/ml		MAp19 ≤ 0.27 µg/ml	
	% (n)	p	% (n)	p	% (n)	p
SIRS	27.7 (13)	0.21	11.1 (5)	0.18	23.9 (11)	0.4
SIRS + MODS	23.4 (11)	**0**.**027**^**a**^	11.1 (5)	0.62	19.6 (9)	0.23
Infection	19.1 (9)	0.85	15.6 (7)	0.40	23.9 (11)	0.34
Fever	29.8 (14)	0.056	28.9 (13)	0.08	30.4 (14)	**0**.**046**^**g**^
LCOS	21.3 (10)	0.3	11.1 (5)	0.25	23.9 (11)	0.11
Liver failure	19.1 (9)	0.17	15.6 (7)	0.81	19.6 (9)	0.23
Renal insufficiency	10.6 (5)	0.65	8.9 (4)	0.98	8.7 (4)	0.87
Death	6.4 (3)	0.47	4.4 (2)	0.24	6.5 (3)	0.54
**complications**	**MASP-1 ≤ 6**.**07 µg/ml**		**MASP-3 ≤ 5**.**18 µg/ml**		**MAp44 ≤ 1**.**81 µg/ml**	
	**% (n)**	**p**	**% (n)**	**p**	**% (n)**	**p**
SIRS	23.9 (11)	0.4	28.3 (13)	0.086	34.8 (16)	**0**.**003**^**c**^
SIRS + MODS	21.7 (10)	0.091	17.4 (8)	0.46	26.1 (12)	**0**.**007**^**d**^
Infection	21.7 (10)	0.6	19.6 (9)	0.91	21.7 (10)	0.59
Fever	23.9 (11)	0.47	21.7 (10)	0.75	21.7 (10)	0.75
LCOS	21.7 (10)	0.26	17.4 (8)	0.82	26.1 (12)	**0**.**038**^**e**^
Liver failure	19.6 (9)	0.23	13.0 (6)	0.81	21.7 (10)	0.087
Renal insufficiency	10.9 (5)	0.67	10.9 (5)	0.66	21.7 (10)	**0**.**001**^**f**^
Death	6.5 (3)	0.54	17.4 (8)	**0**.**016**^**b**^	13.0 (6)	0.23

^a^OR = 2.59, 95% CI (1.09–6.12); ^b^OR = 3.42, 95% CI (1.2–9.73); ^c^OR = 3.15, 95% CI (1.46–6.8); ^d^OR = 3.13, 95% CI (1.32–7.38); ^e^OR = 2.35, 95% CI (1.03–5.36); ^f^OR = 5.2, 95% CI (1.85–14.62); ^g^OR = 2.17, 95% CI (1–4.69).

### Ficolin-3

Median pre-operative ficolin-3 concentration did not differ significantly between the group of patients who were discharged from the hospital with no post-operative complications and any group experiencing any of the complications analyzed (Table 3).

However, low ficolin-3 was more common among patients suffering from SIRS + MODS (p = 0.027, Table 4). Yet within this (SIRS + MODS) subgroup, those who died had significantly higher pre-operative ficolin-3 levels than survivors [medians: 14.8 µg/ml (n = 11) *vs*. 8.4 µg/ml (n = 16); p = 0.03]. Indeed, patients who developed SIRS and MODS with ficolin-3 levels ≤ 25^th^ percentile had generally a much better prognosis [91% survival *vs*. 37%; OR = 16.7; 95% CI (1.7–164.9); p = 0.007]. Conversely, an adverse effect was observed for patients with ficolin-3 ≥75^th^ percentile compared with those <75^th^ percentile: 20% *vs*. 82% survival [OR = 0.05; 95% CI (0.007–0.39); p = 0.003]. These findings prompted us to perform a ROC analysis, to find the best discriminating value in relation to survival. The best fit was found to be 12.7 µg/ml (AUC = 0.75; sensitivity 90.9%; specificity 75%). Using this value, it was calculated once again that low ficolin-3 concentrations are associated with lower mortality in patients who developed SIRS and MODS [92% survival compared with 29% among patients with ficolin-3 above 12.7 µg/ml; OR = 30.0; 95% CI (2.9–313.7); p = 0.001].

The 12.7 µg/ml cut-off also has prognostic value when any post-operative complication co-exists [survival: 94% *vs*. 73% when compared with patients without any post-operative complications; OR = 5.9 95% CI (1.2–28.4); p = 0.02], but not for the patient cohort in total (96% *vs*. 88% survival; p = 0.11).

## Discussion

Post-bypass systemic inflammatory response and other complications are still a problem in cardiac surgery. The incidence of post by-pass SIRS in pediatric patients is estimated to be between 8.9 and 30.5%^31,32^. It often has a fatal outcome and is associated with organ injury, longer ICU and total hospital stays. Complement activation has been considered one of the key mechanisms responsible for the development of post-CPB inflammatory response. In the last 2 decades, the significance of the lectin pathway has been taken into account. Our previous reports evidenced MBL-dependent LP activation during cardiac surgery using cardiopulmonary bypass and the contribution of this phenomenon to the development of post-operative SIRS. We observed also that *MBL2* genotypes and MBL pre-operative serum levels correlate with the risk of various post-bypass complications^5,6^. Furthermore, we found deposition of MBL and ficolins as well as deposition of C4 activation products on the surface of polyurethane tubing routinely used for CPB^33^. Here we reported data concerning other lectin pathway-associated proteins: MASP-1 (crucial for the initiation of the cascade), MASP-2 (responsible for C4 cleavage) as well as MASP-3, non-proteolytic MAp44 and MAp19 (as regulatory factors) as well as ficolin-3 (the most abundant LP-related PRM). It should be also stressed that afore-mentioned MASP cross-talk with alternative pathway activation and/or coagulation cascade/kinin system might contribute to amplification of some adverse effects.

The high expression of MAp44 mRNA in the heart and its significance for cardiac development provokes particular attention^14,34,35^. Our data demonstrated that individuals with low pre-operative MAp44 more frequently suffered from such post-operative complications as SIRS, MODS, LCOS or kidney failure. For MAp19, similar associations were found in children older than 1 year but not in infants. That suggests MAp44 (and MAp19 in older patients) acts to prevent injury from excessive complement activation and thus from systemic inflammation in response to major surgery. Those findings correspond to some extent with significant inverse correlations of MAp44 and MAp19 with such parameters of disease severity as VIS or BAS in older children (Table 2). Therefore, deficient individuals may be at a higher risk of development of afore-mentioned complications and non-enzymatic members of the MASP family might be considered as their potential biomarkers. Generally, results reported here confirmed our previous conclusion that LP activation contributes to development of post-operative SIRS^5,6^. As no association of MASP-2 concentration with postoperative complications or outcome was found, the key role of pattern-recognition molecules (collectins, ficolins) and/or regulatory factors (MAp44, MAp19, MASP-3) or its direct activator (MASP-1, see below) may be supposed. It should be stressed that MASP-2 levels are apparently less variable compared with other tested proteins (as well as MBL^5,6^) therefore even relatively low concentration may be sufficient for as active lectin pathway as allowed by the level of associated PRM and/or other MASP family members. That conclusion is additionally supported by previously published evidence that low activity of MBL-MASP-2 complexes (strongly correlating with MBL concentration and MBL-MASP-1 activity) offer protection against LCOS, SIRS, renal insufficiency and multi-organ failure^5,6^. Furthermore, pre-operative MASP-2 levels did not correlate significantly with MBL-MASP-2 activities (not shown).

According to the afore-mentioned report^6^, low pre-operative serum activity of MBL-MASP-1 complex protected patients from post-operative fever and SIRS but were associated with a higher risk of hospital infections. Our current evaluation of total serum MASP-1 concentration has not shown simply corresponding associations. However, children with the highest levels of MASP-1 (≥11.68 μg/ml, corresponding to the 90^th^ percentile) had a poorer survival rate than those with lower MASP-1. It might be speculated that when a patient experiences severe complications (especially SIRS + MODS), high concentration of this protease (as in the case pattern-recognition molecule, ficolin-3) contributes to the amplification of adverse effects of activation of complement, coagulation and kinin systems. Therefore, high pre-operative levels of both factors involved in the initiation of the LP cascade might enhance the risk of life-threatening events. In parallel, relatively high MAp44 concentrations in some patients (due to significant correlation with MASP-1) seem not to be enough to be protective. In contrast, low MASP-3, another product of the *MASP1/3* gene possibly involved in regulation of complement activation, was associated with high mortality. Previously, low MASP-3 on admission of pediatric patients to intensive care unit was associated with new hospital infections and prolonged stay at the ward^36^. We have not found such an influence of that enzyme in the case of patients suffering from CHD, although its concentration correlated reversely with VIS (Table 2).

Earlier, Frauenknecht *et al*.^37^ found plasma MASP-1 concentrations higher and MASP-2 lower in patients with myocardial infarction (MI) than in healthy controls. MASP-3 and MAp44 did not differ significantly^37^. Next, Holt *et al*.^38^ reported significantly higher plasma MAp44, MASP-1, and MASP-3 concentrations in MI, although no association with short-term outcome was observed. However, low MAp44 level predicted higher risk of death in renal transplant recipients while neither MASP-3 nor MAp19 concentrations were associated with patients’ survival^39^.

Ficolin-3-dependent lectin pathway activation during CPB was originally reported by Hein *et al*.^40^. Earlier, Xuan *et al*.^41^ reported lower ficolin-3 concentrations in newborns with tetralogy of Fallot (TOF) and ventricular septum defect (VSD) than in healthy controls. The lack of age-matched control group of children without CHD in our study makes similar comparison impossible. It has to be emphasized that aim of our study (which was investigation of associations of selected proteins with post-operative complications but not CHD itself) did not assume comparisons with healthy controls. On the other hand, we observed relatively higher ficolin-3 concentrations in TOF and VSD patients than in other types of CHD (not shown). Our most important findings concerning that protein are perhaps an association of its low pre-operative serum concentration with the development of SIRS and MODS and, secondly, its potential predictive value. It is not obvious why lower levels of a factor involved in complement activation contribute to excessive inflammatory response finally resulting in multiorgan failure. Although low ficolin-3 accompanied by high C3a level was associated with chronic heart failure^42^, that cannot be directly related to our results. It has been suggested that altered self-structures in the failing heart may bind ficolin-3, leading to its lowered plasma levels and induction of complement activation^42^. On the other hand, according to our data, in patients who developed SIRS followed by MODS, lower pre-operative serum ficolin-3 concentration seems to be beneficial. After further, possibly independent investigation, that protein might be considered as prognostic factor in the cases of severe complications after CPB. Our results (although from a relatively small number of pediatric patients) suggest that a suitable cut-off level would be 12.7 μg/ml. Previously, high ficolin-3 (as well as MASP-2) levels in plasma were suggested to predict a fatal outcome after severe traumatic brain injury^43,44^. Previously, ficolin-3 has been considered an anti-microbial agent^45^, however we did not observe a significant difference between its median concentrations in patients who developed post-operative infections and those who did not. That might support our earlier hypothesis that the crucial role of that factor may be in controlling normal (commensal) flora rather than protection from obligatory pathogens (including agents of hospital infections).

Although concentrations of ficolin-3 and MASP family proteins (with an exception for MASP-3) have been reported to differ between males and females^28,46^ no such differences were seen in our pediatric patients (not shown). It may be speculated that such differences become evident during adolescence.

To summarize, our data add considerably to the knowledge concerning involvement of complement activation *via* the lectin pathway in development of post-CPB complications. Possible interaction with other endogenous cascades, make the problem more complicated and data difficult to interpret, but create additional questions to be answered. Of immediate interest are our findings of (i) low preoperative MAp44 and various post-operative complications and (ii) high preoperative ficolin-3 and increased mortality. These promising prognostic associations warrant independent confirmation, perhaps by a large multi-centre study.

## Supplementary information

Supplementary Dataset 1
