# Supplementary material for: Factors involved in initiation and regulation of complement lectin pathway influence postoperative outcome after pediatric cardiac surgery involving cardiopulmonary bypass
Source: Sci Rep. 2019 Feb 27;9:2930. doi: 10.1038/s41598-019-39742-w (PMC6393526; doi:10.1038/s41598-019-39742-w)
Supplement: Supplementary file 1 — Supplementary Dataset 1 [file 41598_2019_39742_MOESM1_ESM.docx]

**SUPLEMENTARY MATERIAL**

**Factors involved in initiation and regulation of complement lectin pathway predict postoperative outcome after pediatric cardiac surgery involving cardiopulmonary bypass**

Mateusz Michalski^1^, Izabela Pągowska-Klimek^2^, Steffen Thiel^3^, Anna S. Świerzko^1^, Annette G. Hansen^3^, Jens C. Jensenius^3^, Maciej Cedzyński^1^*

^1^Laboratory of Immunobiology of Infections, Institute of Medical Biology, Polish Academy of Sciences, Lodz, Poland; ^2^Department of Pediatric Anesthesiology and Intensive Care Medical University of Warsaw; ^3^Department of Biomedicine, Aarhus University, Aarhus, Denmark

**Table S1.** Cut-off values for pre-operative serum concentrations chosen for analyses

| Protein | Cut-offs (µg/ml) | |
| --- | --- | --- |
|  | 25^th^ percentile | 75^th^ percentile |
| Ficolin-3 | 10.1 | 21 |
| MASP-1 | 6.07 | 9.72 |
| MASP-2 | 0.18 | 0.33 |
| MASP-3 | 5.18 | 7.50 |
| MAp44 | 1.81 | 2.57 |
| MAp19 | 0.27 | 0.39 |

**Table S2.** Correlations between serum concentrations of lectin pathway-associated proteins with age of patients and with each other (significance in parentheses).

|  | Age | Ficolin-3 | MASP-1 | MASP-2 | MASP-3 | MAp44 |
| --- | --- | --- | --- | --- | --- | --- |
| MAp19 | r=0.13  p=0.07 | **r=0.19**  **p=0.009** | r=0.03  p=0.65 | r=0.13  p=0.09 | **r=0.22**  **p=0.003** | **r=0.24**  **p=0.001** |
| MAp44 | **r=-03**  **p< 0.0001** | **r=0.2**  **p=0.006** | **r=0.39**  **p<0.0001** | r=-0.04  p=0.64 | **r=0.21**  **p=0.005** |  |
| MASP-3 | **r=0.25**  **p=0.001** | r=0.14  p=0.052 | r=-0.09  p=0.21 | r=0.05  p=0.54 |  |  |
| MASP-2 | r=0.13  p=0.08 | **r=0.29**  **p=0.0001** | r=0.01  p=0.90 |  |  |  |
| MASP-1 | **r=-0.24**  **p=0.001** | **r=0.17**  **p=0.02** |  |  |  |  |
| Ficolin-3 | r=-0.007  p=0.92 |  |  |  |  |  |
